# Supplementary material for: Using a One Health approach to prioritize zoonotic diseases in China, 2019
Source: PLoS One. 2021 Nov 19;16(11):e0259706. doi: 10.1371/journal.pone.0259706 (PMC8604330; doi:10.1371/journal.pone.0259706)
Supplement: Attachment — Submitted filename: Response to Reviewers.doc [file pone.0259706.s002.doc]

**Response to Journal and Reviewers**

**Journal Requirements**

Please review your reference list to ensure that it is complete and correct. If you have cited papers that have been retracted, please include the rationale for doing so in the manuscript text, or remove these references and replace them with relevant current references. Any changes to the reference list should be mentioned in the rebuttal letter that accompanies your revised manuscript. If you need to cite a retracted article, indicate the article’s retracted status in the References list and also include a citation and full reference for the retraction notice.

**Response to Journal**

Thanks for providing the requirements. Accordingly, the reference list has been reviewed and revised as follows:

1.Reference [3] is revised to include complete information on line 346-347.

2.Reference [30] is provided with updated URL on line 417, to replace formerly invalid URL.

3.All URL of the reference has been checked for validity, and ‘Last accessed MMMDD,YYYY’ was updated for references [3][10][13][18][25][28][29][30] .

4.No reference has been found retracted, by searching references in <https://pubmed.ncbi.nlm.nih.gov/>.

**Reviewer 1**

The authors addressed most of our comments. Explanations were added for the categories and inclusions of workshop participants as well as the formation of the initial zoonotic disease list. Statements were added for expanding upon weighting and ranking methods. These additions improved the methods section. An explanation was added concerning COVID-19, the timing of the 2019 workshop, and considerations for the future. This addition helps readers understand the lower ranking of SARS.

**Response to Reviewer 1**

Thanks for reviewer’s comment. We deeply appreciate your review on the paper. We have studied the comments carefully and revised the manuscript that takes all the points into account. The following addresses each of the comments item by item. We would like to express our appreciation for your professional comments during each revision, which have really helped the paper improved much.

**Minor edits**
Q1:

An explanation of the Baidu index was added, although more language regarding the bias of this approach would be useful to readers.
The authors’ reply to comment 1 could be incorporated into the manuscript text regarding participation of certain provinces and geographic locations. Including an explanation about provincial participation and potential biases would strengthen the methods section.

A1:

Thanks for your comments. Certain provinces were added to geographic locations on line 100-101,

“The eight provinces represent the primary ecological and socio-demographic regions in the east (Jiangsu and Shandong), northwest (Qinghai, Ningxia, and Gansu ) and southwest (Guangxi, Sichuan and Yunnan) of China respectively. ”

Thanks for your suggestion of incorporating our reply to previous comment 1 into this revision about provincial participation and potential biases . As the example of our previous reply, though staffs from Heilongjiang and Hunan Province were unnable to attend the workshop, Yin Wenwu (who attended) and his team from China CDC worked closely with these provinces, the high-risk province for rabies and anthrax respectively.

Accordingly, it is revised on line 101-103:

“Not all provinces were able to participate. However, national managers participating in the workshop were able to provide input on behalf of the human, animal, and environmental health sector staff in the provinces unable to attend. ”

Q2:
Table 1: The number of agencies is less than the number of participants. Could you add the number of participants per agency?

A2:

Thanks for your comments. Accordingly to the comments, the number of participants has been added to each agency in table 1.

**Voting Members (No. participants)**

***National Level***

National Institute for Communicable Diseases Control and Prevention, China CDC (2)

National Institute of Parasitic Diseases (NIPD), China CDC (1)

Division of Infectious Disease, China CDC (1)

China Animal Disease Control Centre (1)

National Research Center for Wildlife Diseases (1)

***Eastern Region***

Jiangsu Provincial CDC (2)

Shandong Provincial CDC (1)

Jiangsu Academy of Agricultural Sciences, Jiangsu Society of Animal Husbandry and Veterinary Medicine (1)

Shandong Provincial Animal CDC (1)

***Northwestern Region***

Qinghai Provincial CDC (3)

Gansu Provincial CDC (1)

Ningxia Hui Autonomous Region CDC (1)

Qinghai Animal CDC (2)

Lanzhou Veterinary Research Institute, Chinese Academy of Agricultural Sciences (1)

Qinghai Academy of Animal Science and Veterinary Medicine (3)

***Southwestern Region***

Yunnan Provincial CDC (1)

Guangxi University of Chinese Medicine, School of Public Health and Management (1)

Sichuan Provincial CDC (1)

Guangxi Zhuang Autonomous Region Animal CDC (1)

Yunnan Provincial Institute of Endemic Disease Prevention and Control (1)

Sichuan Provincial Animal CDC (1)

Q3:

The previous comment concerning terminology of diseases versus infections was only partially addressed. The authors replied that the terminology was used for consistency with previous OHZDP workshops. Changing the terminology to correctly identify infections or diseases should be implemented. At the very least, a statement regarding this ‘consistency’ of terminology should be included.

A3:

Your understanding for the consistency with previous OHZDP workshops is much appreciated. A statement regarding this ‘consistency’ is included in method section on line 129-131 as,

“For consistency with publications resulting from previous workshops, we use the term zoonotic diseases rather than zoonotic infections. The term highlights that these priority zoonotic infections are capable of causing disease.”

Q4:
In the discussion, limitations or implications of use of the chosen criteria (not just social impact) should be added.

A4:

Thanks for reviewer’s comments, the limitations of using chosen criteria is important and should be added. The method of One Health Zoonotic Disease Prioritization has been utilized in more than 25 locations, including USA, Uganda, Kenya, Kenya, etc [13-17]. Much experiences have been accumulated by the workshop and are inherited by the workshop advisor. The national level workshop did not aim to address all possible zoonotic disease prioritization criteria since the OHZDP methods focus on the top five criteria of greatest concern.Participants discussed many different criteria during the workshop and agreed on the five criteria most likely associated the potential zoonotic disease threat in the country.

Each criteria has a corresponding question, which is required an ordinal or multinomial answer to measure the diseases. When deciding criteria, the participants bear in mind to cover as much diseases as possible, but still a few disease has no answer to certain criteria’s question. Taking criteria “Economic impact” as example, livestock production loss is the question used for measurement. If livestock production loss is not available, case fatality rate and mortality were used as proxies; if China-specific data is not available, global data is used instead (see line 454-456, footnote * of Table 3). Future workshops – including those conducted at the regional or provincial level - could develop and apply more targeted and specific criteria for identifying a local list of priority zoonotic diseases.

According revision is added on line 304-310.
